# Supplementary material for: Hydrogen Protection Boosts the Bioactivity of Chrysanthemum morifolium Extract in Preventing Palmitate-Induced Endothelial Dysfunction by Restoring MFN2 and Alleviating Oxidative Stress in HAEC Cells
Source: Antioxidants (Basel). 2023 Apr 28;12(5):1019. doi: 10.3390/antiox12051019 (PMC10215833; doi:10.3390/antiox12051019)
Supplement: Supplementary file 1 [file antioxidants-12-01019-s001.zip › antioxidants-2358184-supplementary.pdf]

Supplementary Table S1. Primary antibodies used for western blot analysis.

Target Protein	Company (Cat. No.)	Molecular Weight (KD)	Dilutions
eNOS	Abcam (ab199956)	140	1:1000
p-eNOS (S1177)	Abcam (ab215717)	140	1:1000
DRP1	Santa Cruz (sc-271583)	82	1:500
MFN1	Santa Cruz (sc-166644)	86	1:500
MFN2	Santa Cruz (sc-100560)	86	1:500
OPA1	BD (612606)	90	1:1000
ATP5A	Invitrogen (459240)	54	1:1000
MTCO1	Invitrogen (459600)	37	1:1000
UQCRC1	Invitrogen (459140)	50	1:2000
SDHB	Invitrogen (459230)	30	1:2000
NDUFS3	Invitrogen (459130)	26	1:1000
NRF2	Santa Cruz (sc-365949)	61	1:500
KEAP1	Cell Signaling (46778S)	60	1:1000
NQO1	Santa cruz (sc-376023)	31	1:500
CATALASE	Cell Signaling (14097S)	60	1:1000
SOD1	Santa cruz (sc-101523)	22	1:500
SOD2	Santa cruz (sc-137254)	23	1:500
GAPDH	Cell Signaling (2118S)	37	1:3000
HISTONE-H3	Abcam (ab1791)	17	1:1000

Supplementary Table S2. Primers used for qPCR.

Target Gene	Forward (5´-3´)	Reverse (5´-3´)
Human *β-ACTIN*	CATGTACGTTGCTATCCAGGC	ACATGACTGGGTCCAAGTTGT
Human *IL6*	TTTTGTACTCATCTGCACAGC	GGATTCAATGAGGAGACTTGC
Human *MMP1*	ACGCCAGATTTGCCAAGAG	TTGACCCTCAGAGACCTTGGT
Human *CHOP*	GGAAACAGAGTGGTCATTCCC	CTGCTTGAGCCGTTCATTCTC
Human *XBP1*	CCCTCCAGAACATCTCCCCAT	ACATGACTGGGTCCAAGTTGT
Human *GRP78*	GAAAGAAGGTTACCCATGCAGT	CAGGCCATAAGCAATAGCAGC
Human *GRP94*	GCTGACGATGAAGTTGATGTGG	CATCCGTCCTTGATCCTTCTCTA
Human *NRF2*	TCCAGTCAGAAACCAGTGGAT	GAATGTCTGCGCCAAAAGCTG
Human *SOD2*	GCAGTGTGCGGCACCAGCAG	TCCCTTGGCCAACGCCTCCT
Human *18S rRNA*	CAGCCACCCGAGATTGAGCA	TAGTAGCGACGGGCGGTGTG
Human *D-LOOP*	CAGTACCTAACAAACCCACA	GAGGTCGTAAACCCTATTGT

Supplementary Table S3. siRNAs used for transfection.

Target Gene	Sense (5´-3´)	Antisense (5´-3´)
Human *MFN2*	GGCCAAACAUCUUCAUCCUTT	AGGAUGAAGAUGUUUGGCCTT
Human *SOD2*	GGGUUGGCUUGGUUUCAAUTT	AUUGAAACCAAGCCAACCCTT

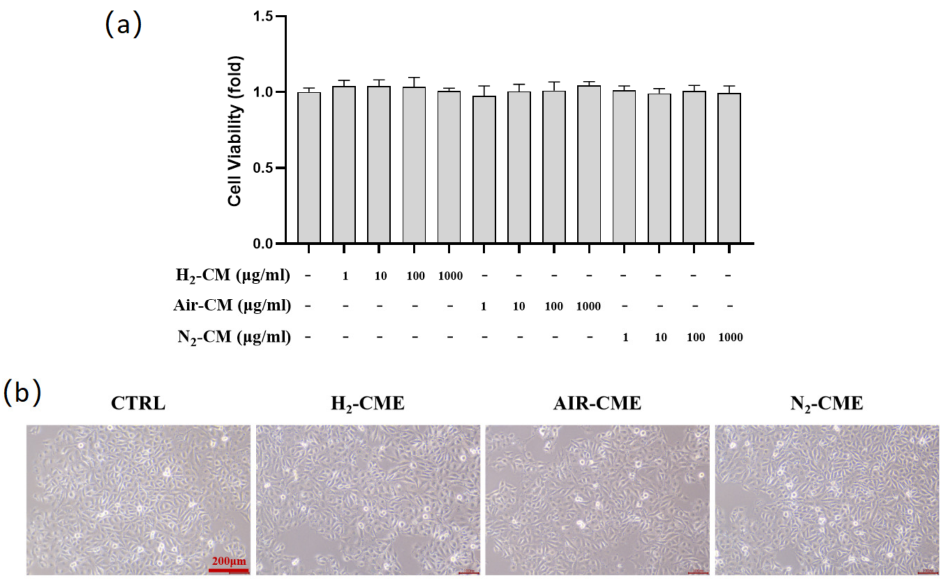

Supplementary Figure S1. Effects of CME on cell viability and morphology in HAEC cells.
(a) HAECs were treated with CME for 24 hours. Cell viability was measured by MTT assay. n= 6. (b) HAECs were treated with 1000 μg/ml CME for 24 h. Observation of cell morphology using an inverted microscope. Magnification: ×10. Scale bar = 200 μm.

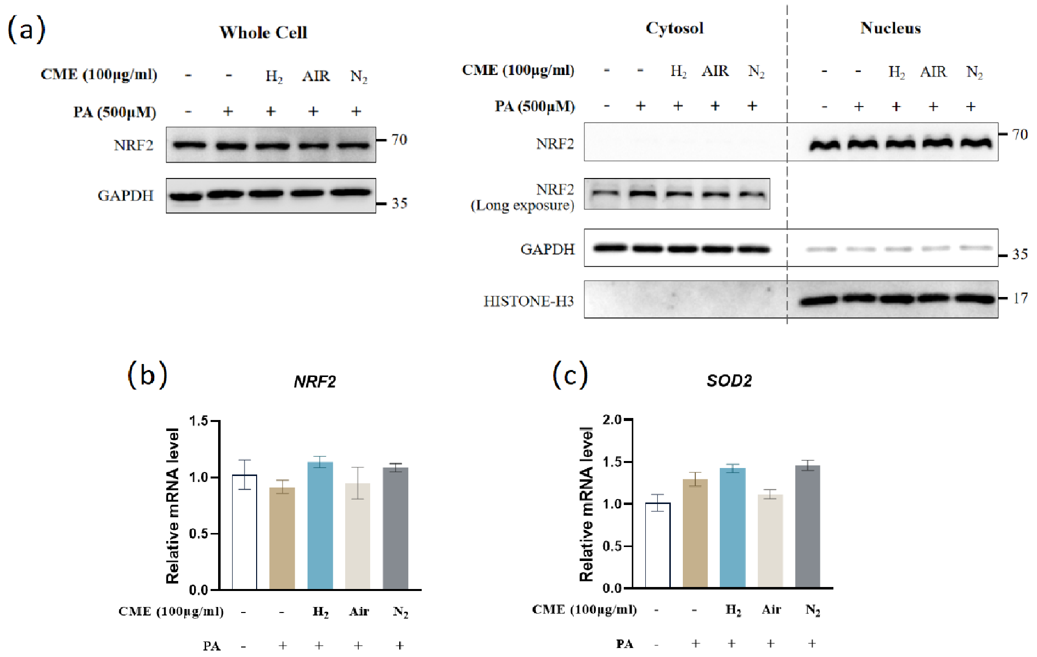

Supplementary Figure S2. Nuclear translocation of NRF2 and mRNA levels of *NRF2* and *SOD2* in response to CME pretreatment and PA challenge in HAEC cells.

HAECs were pretreated with 100 μg/ml CME for 24 h, followed by 500 μM PA or BSA treatment for another 24 h.

(a) Western blot analysis of nuclear translocation of NRF2.

(b-c) mRNA level of NRF2 (b) and SOD2 (c) was measured by qRT-PCR. n=4.
